# Thermodynamic analysis of regulation in metabolic networks using constraint-based modeling

**DOI:** 10.1186/1756-0500-3-125

**Published:** 2010-05-05

**Authors:** Srinath Garg, Laurence Yang, Radhakrishnan Mahadevan

**Affiliations:** 1 Department of Chemical Engineering and Applied Chemistry, University of Toronto, Ontario-M5S3E5, Canada; 2 Institute of Biomaterials and Bioengineering, University of Toronto, Ontario-M5S3E1, Canada

## Abstract

**Background:**

*Geobacter sulfurreducens *is a member of the *Geobacter *species, which are capable of oxidation of organic waste coupled to the reduction of heavy metals and electrode with applications in bioremediation and bioenergy generation. While the metabolism of this organism has been studied through the development of a stoichiometry based genome-scale metabolic model, the associated regulatory network has not yet been well studied. In this manuscript, we report on the implementation of a thermodynamics based metabolic flux model for *Geobacter sulfurreducens*. We use this updated model to identify reactions that are subject to regulatory control in the metabolic network of *G. sulfurreducens *using thermodynamic variability analysis.

**Findings:**

As a first step, we have validated the regulatory sites and bottleneck reactions predicted by the thermodynamic flux analysis in *E. coli *by evaluating the expression ranges of the corresponding genes. We then identified ten reactions in the metabolic network of *G. sulfurreducens *that are predicted to be candidates for regulation. We then compared the free energy ranges for these reactions with the corresponding gene expression fold changes under conditions of different environmental and genetic perturbations and show that the model predictions of regulation are consistent with data. In addition, we also identify reactions that operate close to equilibrium and show that the experimentally determined exchange coefficient (a measure of reversibility) is significant for these reactions.

**Conclusions:**

Application of the thermodynamic constraints resulted in identification of potential bottleneck reactions not only from the central metabolism but also from the nucleotide and amino acid subsystems, thereby showing the highly coupled nature of the thermodynamic constraints. In addition, thermodynamic variability analysis serves as a valuable tool in estimating the ranges of Δ_r_G' of every reaction in the model leading to the prediction of regulatory sites in the metabolic network, thereby characterizing the regulatory network in both a model organism such as *E. coli *as well as a non model organism such as *G. sulfurreducens*.

## Introduction

*Geobacter sulfurreducens *is a well studied representative of the *Geobacteraceae *family of microorganisms. The *Geobacter *species are of interest due to their role in carbon and mineral recycling and their capacity to harvest electricity from organic wastes. Furthermore, previous studies in a uranium contaminated aquifer in Rifle, CO have shown a significant decrease in the levels of hexavalent U(VI) when acetate was injected in the subsurface. The injection of acetate was followed by a bloom in the population of *Geobacter *species in the subsurface. *G. sulfurreducens *is the most commonly studied species as its complete genome has been sequenced and protocols for whole genome microarrays and proteomics are available. Furthermore, recent sequencing efforts have provided us with a wealth of information regarding the genetic content of various organisms [1]. In order to better understand the metabolism of *G. sulfurreducens*, a genome scale *in silico *model was constructed utilizing the wealth of omics data [2]. This model was used to correctly predict growth phenotypes for knockouts under varying conditions and was able to quantify the effect of global proton balance on the biomass yield during Fe(III) reduction [2]. Furthermore, recent studies have shown that the constraint-based model can successfully account for changes in the metabolic network in response to environmental perturbations and thus predict the condition specific differences in microbial metabolism [3]. Previous studies have shown that a fundamental understanding of regulation is required for accurately predicting the metabolic response of *E. coli *to both environmental and genetic perturbations. Hence, characterizing the reactions that are subject to regulation in the metabolic network of *G. sulfurreducens *is important for optimizing the practical applications of *G. sulfurreducens*. In this report, we first validate the utility of a thermodynamics based metabolic flux analysis model (TMFA) to predict regulatory sites by comparing model predictions with the corresponding expression data for *E. coli*. Subsequently, we present the implementation of a TMFA model for the genome-scale metabolic network of *G. sulfurreducens *in order to eliminate internal flux cycles and to generate thermodynamically feasible flux distributions. This approach takes into account both standard Gibbs free energy change as well as the concentration/activity dependent free energy change associated with the mixing term, thereby effectively coupling the metabolite concentrations and the Gibbs free energy change. Based on the standard free energy change of reactions and the defined molar concentration ranges, the ranges in free energy change of every reaction in the model was estimated by thermodynamic variability analysis (TVA) which involved maximizing and minimizing the standard free energy change of each reaction in the metabolic network of *G. sulfurreducens *at optimal growth conditions.

## Materials and methods

### Flux balance analysis and extension to TMFA

The mass balance constraint described by the following equation enforced by Flux balance analysis (FBA) forms the basis of the TMFA algorithm.

In the above equation, S is the Stoichiometric matrix with dimensions (m × n) where m and n refer to the total number of metabolites and fluxes, respectively, in the metabolic network of an organism and c represents a biological objective function. In the case of *G. sulfurreducens*, this matrix has the dimension of 542 × 611. FBA has been used in the past to address the effect of gene knockouts, predicting the ability of a cell to produce a biochemical or to predict the maximum possible growth yield of a cell. Hence, coupling of thermodynamic constraints based on the second law of thermodynamics with the mass balance constraint of the FBA limits fluxes through futile cycles while still allowing fluxes through feasible pathways.

### Formalism of the TMFA algorithm

We used the TMFA algorithm previously developed by Henry and co-workers [4] to predict regulatory targets and metabolite activity ranges in the *E. coli *metabolic network. Briefly, the TMFA algorithm complements the mass balance constraints of FBA with mixed integer type thermodynamic constraints and is reproduced below for the sake of continuity:

In the above formulation, Z_i _is a binary decision variable that can either assume values 0 or 1 depending on the Gibbs free energy change of a particular reaction. In some cases, the free energy of formation of certain metabolites cannot be estimated by the group contribution method due to the unique molecular substructures associated with them. Such reactions (a total of L) are typically lumped together so as to eliminate those metabolites whose standard free energy change is unknown. Here, y_i _represents whether the lumped reaction is active (0) or not (1) and α_i, j _represents whether the j^th ^reaction participates in the i^th ^lumped reaction and equals 1 when the j^th ^reaction is a part of the lumped reaction. Consequently, the final two constraints in the equation indicate the thermodynamic feasibility constraint as applied to these lumped reactions.

### Estimating the standard free energy changes of reactions

Implementing the TMFA algorithm requires the knowledge of the standard free energy change of every reaction in the metabolic network to be either determined or estimated. Since experimental values are available for only a small number of reactions in the literature, the improved group contribution method [5] was used to determine the standard free energy change of every reaction in the model (see Additional File 1: Supplemental Table S1). In order to account for the error in fitting the group contribution energy values, the error in the energy contribution of molecular substructure was allowed to vary within two standard errors [4].

### Thermodynamic variability analysis (TVA)

The free energy ranges of reactions were estimated using thermodynamic variability analysis [4], which is similar to the flux variability analysis, except that in this case the standard free energy change of every reaction in the model is maximized and minimized to find the range of free energies for every reaction in the model under optimal growth conditions. This analysis is performed subject to both mass balance and thermodynamic constraints and results in thermodynamically allowable ranges for the free energies for every reaction in the network. For the analysis of *G. sulfurreducens *metabolism, simulations were done for growth on acetate as the donor and fumarate as the electron acceptor. For growth on acetate as the energy source and fumarate as the sole electron acceptor, the uptake rate for acetate was set to 5 mmol/gdw hr and the uptake rate of fumarate was set to 25 mmol/gdw hr, so that these uptake rates were consistent with experimentally determined values. For growth conditions involving acetate as the energy source and fumarate as the sole acceptor, the optimal growth rate was forced to be 0.055 1/hr [2].

### Correlation with gene expression data

The thermodynamic ranges calculated by TVA were used to identify reactions that were 1) bottleneck reactions which have free energy changes close to zero, and 2) candidates for regulation which have free energy change ranges that are either positive or negative and do not span zero (equilibrium condition). The thermodynamic bottleneck reactions were identified by utilizing the free energy change values reported previously for near equilibrium systems as the basis [6]. In order to validate the model prediction of regulation, the gene expression fold changes that are significant (defined based on datasets, where the coefficient of variation <0.2) corresponding to the predicted regulatory candidates were calculated. Gene expression data was obtained from previously published studies that investigated different microarray experiments under several environmental and genetic perturbations. For the case of *G. sulfurreducens*, we investigated twenty one different microarray experiments (See Additional File 2: Supplemental Table S2) and calculated the gene expression fold change for each gene associated with the thermodynamic bottleneck reactions and the reactions that are subject to regulatory control. For the case of *E. coli*, we obtained gene expression data sets from a public database, namely, http://genexpdb.ou.edu/index.php)- *E. coli *community gene expression database (GenExpDB), in which gene expression data for over 800 experiments were reported.

## Results

### Thermodynamic Metabolic Flux Analysis of *E. coli*

#### Comparison of the predicted regulatory sites of the E. coli metabolic network with gene expression data

The magnitude of free energy change of a biochemical reaction dictates if the reaction is subject to regulation. Typically, biochemical reactions that operate close to equilibrium are not transcriptionally regulated, while reactions that operate far from equilibrium are insensitive to perturbations in the metabolite concentrations and can therefore be subject to regulation as the flux through such reactions can be manipulated by enzyme regulation alone. In contrast, thermodynamic bottlenecks are reactions that operate close to equilibrium. Consequently, even minor perturbations in the concentration of the reactants or products can force the net flux through these reactions to zero, thereby blocking them or changing their directions. Reactions identified using TVA for which Δ_r_G' is strictly negative were classified as candidates for regulation as these reactions cannot reach equilibrium under the activity ranges studied. Thus, based on the above criteria, we used the TVA analysis to predict both bottleneck reactions and candidates for regulation based on the observation that reactions that are irreversible under most conditions are likely to be regulated [4,7,8].

Beard, Qian and co-workers were pioneers in conducting energy balance analysis using non linear optimization to eliminate flux cycles generated by FBA in *E. coli *[9-11]. Kummel *et al. *further refined this method to quantitatively analyze metabolomic data sets in *E. coli *and *S. cerevisiae *[7], while Henry and co-workers [4] extended the method further to the genome scale to identify feasible metabolite activity ranges, bottleneck reactions, and reactions subject to regulatory control in the genome-scale network of *E. coli*. However, we note that, in previous studies, the identified bottleneck reactions and regulatory sites were not compared against gene expression data and were not thus comprehensively validated. It is in this aspect that our present work differs significantly as we have analyzed microarray data from a central repository with over 800 experimental datasets.

**Figure 1:**
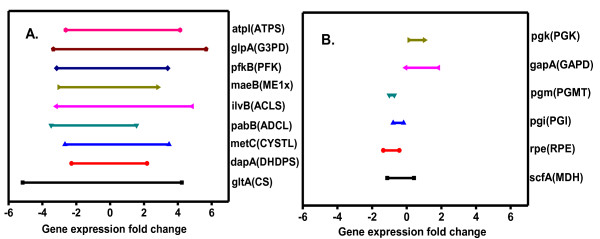
**Comparison of filtered gene expression fold changes across different conditions (environmental and genetic perturbations) for model predicted reactions subject to regulation (A) and thermodynamic bottlenecks (B) in *E. coli***. Reaction abbreviations are enclosed within parentheses.

In order to validate predicted regulatory targets and bottleneck reactions in *E. coli*, we identified the range of gene expression for those reactions that were previously predicted [4,6] to be operating close to equilibrium (thermodynamic bottleneck reactions) and those reactions that operated far from equilibrium (reaction subject to regulatory control) in *E. coli *(Figure 1A &1B and Additional File 2: Supplemental Tables S3 & S4). As bottleneck reactions operate close to thermodynamic equilibrium, they are not likely to be regulated at the transcript level and correspondingly, the range of the gene expression fold change is expected to be narrower relative to the putative regulatory sites. The results of the comparison of ranges of gene expression data for thermodynamic bottleneck reactions and putative regulatory sites are shown in Figure 1. It was observed that the expression fold change for genes corresponding to the thermodynamic bottleneck reactions (Figure 1B) were narrower when compared to the reactions subject to regulatory control (Figure 1A), thereby validating the claim that these reactions were subject to regulation *in vivo *in *E. coli*. To verify if these results were statistically significant, we performed a two sided wilcoxon rank sum test considering the ranges in the reported gene expression fold changes for these two classes of reactions. Our analysis indicated a p value of 3.9960e-04, thereby showing that the medians are statistically significant for the two sample sets.

#### Correlation of predicted bottleneck reactions with exchange coefficients from C13 isotope labeling studies

Since thermodynamic bottleneck reactions operate in the vicinity of equilibrium, they are highly reversible in nature and can consequently have significant forward and reverse fluxes. Thus, we can associate an exchange coefficient (defined as the ratio of the forward to reverse flux assuming the forward flux is greater than the reverse flux) with these reactions. C^13 ^isotope labeling studies can be used to measure the intracellular metabolic fluxes as well as the exchange coefficients [12]. Also, the exchange coefficients represent a measure of reversibility of a reaction and are determined from the experimentally measured mass isotopomer fractions of the amino acids. Hence, for these thermodynamic bottleneck reactions, we decided to evaluate previously published exchange coefficients from C^13 ^isotope labeling experiments in aerobically grown *E. coli *with glucose as the substrate [13]. We found that three of the predicted bottleneck reactions, namely, phosphoglucose isomerase (PGI), glyceraldehyde 3-phoshpate dehydrogenase (GAPD), and malate dehydrogenase (MDH) had statistically significant exchange coefficients (PGI: 0.761, GAPD:0.402, MDH: 0.02), suggesting there was a significant forward and reverse flux at these predicted bottleneck reactions and that these reactions operate close to equilibrium *in vivo*. Consequently, the utility of using TMFA for the prediction of regulatory sites and bottleneck reactions was validated for genome-scale metabolic network of *E. coli*.

### Thermodynamic Metabolic Flux Analysis of *G. sulfurreducens*

#### Predicted reactions subject to regulatory control in G. sulfurreducens and the corresponding gene expression ranges

**Figure 2:**
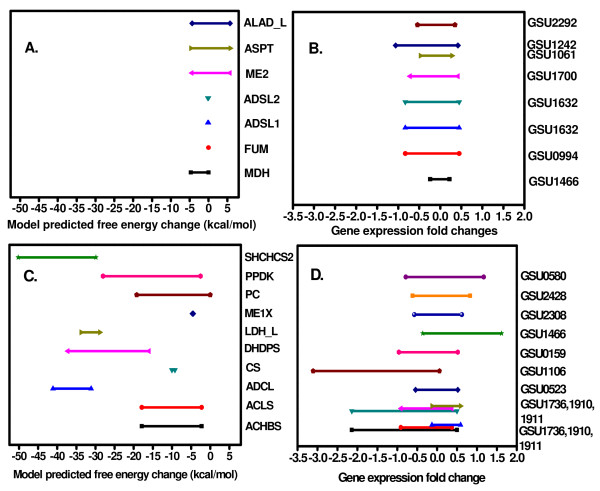
**Comparison of filtered gene expression fold changes across different conditions (environmental and genetic perturbations) and the corresponding ranges of the free energy changes for model predicted reactions subject to regulation (C and D) and thermodynamic bottlenecks (A and B) in *G. sulfurreducens***.

In order to evaluate the potential of the TMFA for the analysis of a relatively poorly characterized organism such as *G. sulfurreducens *compared to *E. coli*, we implemented the TMFA for the genome-scale metabolic network of *G. sulfurreducens*. We subsequently used the TVA to calculate the range of Gibbs free energy change for each reaction in the network resulting in the identification of thermodynamic bottlenecks and putative regulatory sites (Figure 2A and 2C). Furthermore, application of thermodynamic constraints identified three others reactions namely, CYSTL (Cystathionine b-lyase), PPS (Phosphoenol pyruvate synthase) and DXPS (1-deoxy-D-xylulose-5-phosphatesynthase), whose free energy ranges indicate that they were on the threshold of regulation (See Additional File 2: Supplemental Tables S5, S6 & S7).

There were several common reactions among the predicted regulatory and bottleneck reactions for both *E. coli *and *G. sulfurreducens*, suggesting that the flux through these reactions is perhaps regulated in a similar manner across different metabolic networks. For example, malate dehydrogenase (MDH) which was identified to be thermodynamically constrained in *E. coli *is similarly identified as a constrained reaction in the *G. sulfurreducens*. In addition, Cystathionine b-lyase (CYSTL), 4-aminobenzoate synthase (ADCL), acetolactate synthase (ACLS), dihydrodipicolinate synthase (DHDPS), citrate synthase (CS), malic enzyme (ME1x) were predicted to be regulated in *E. coli *and these reactions were identified by TVA to be subject to regulation in *G. sulfurreducens *as well. Of these reactions, CYSTL, DHDPS and ACLS form a linear pathway in both *G. sulfurreducens *and *E. coli*.

We then evaluated the range of gene expression fold changes for the predicted regulatory targets and bottleneck reactions based on previously published microarray data (See Additional File 3: Supplemental Table S8) for *G. sulfurreducens *(Figure 2). These results indicate that the genes corresponding to the predicted regulatory candidates appear to have a broader range of fold changes (Figure 2D) relative to the thermodynamic bottleneck reactions, while the predicted bottleneck reactions have the narrowest range of gene expression (Figure 2B). Furthermore, a two sample t-test assuming un-equal variance suggested a p value of 0.029 that is statistically significant at the 95% confidence level.

#### Prediction of Thermodynamic bottleneck reactions in G. sulfurreducens and the correlation with corresponding exchange coefficients

Thermodynamic variability analysis in the absence of uncertainty about the standard free energy changes identified seven reactions that participate as bottlenecks in the metabolic network of *G. sulfurreducens *(Figure 2A). The analysis was done in the absence of uncertainty as we wanted to focus our analysis on the ranges the free energies could assume when they were influenced by the activities of metabolites alone. In order to validate these predictions, we decided to investigate previously published C^13 ^flux analysis data of *G. sulfurreducens*.

Analysis of C^13 ^labeled flux analysis data revealed exchange coefficients associated with two of the seven identified bottleneck reactions. The exchange coefficient for MDH and FUM were 0.29 and 0.37 respectively [14]. These results show that reactions that have significant forward and reverse fluxes are highly reversible. This results in scrambling of the C^13 ^label at this step. Hence, this observation validates the prediction that these reactions operate close to equilibrium *in vivo*.

## Discussion

This study shows the importance of applying thermodynamic constraints to a genome-scale metabolic model for a rigorous study of the entire metabolic network of *G. sulfurreducens*. It is clear that the application of thermodynamic feasibility constraints to a stoichiometric model results in the expanded applicability of flux balance analysis methods without adversely affecting their accuracy. Application of TVA also identified the potential for genes corresponding to metabolic reactions to be transcriptionally regulated. It is hypothesized that identifying the candidate reactions for regulation can help in identifying modules in metabolic networks, as a key step for regulation in many metabolic pathway is the first step of a linear pathway in a specific module. For example, the reaction CYSTL is the first step of the pathway that produces exclusively methionine and is identified as a candidate for regulation by TVA. In addition to the CYSTL reaction, the simulations also identify ACLS, which is the first step of the valine, leucine and isoleucine metabolism pathway that exclusively produces 2-acetolactate. Yet another example is the DHDPS reaction from the threonine and lysine metabolism pathway that is a first step of the pathway that exclusively produces 2,3-dihydrodipicolinate. Both these reactions, namely DHDPS and ACLS were identified by TMFA algorithm to be subject to regulation. Hence, these results suggest that the regulatory sites identified by the TMFA could point to regulatory modules where the first step of the linear pathway being regulated. These results also suggest that the incorporation of additional experimental metabolomic data under different environmental perturbations would help unravel more unravel more examples of regulatory targets and modules. Additionally, applications of thermodynamic constraints have also shown to help in identifying thermodynamic bottlenecks in the metabolic network of *G. sulfurreducens*. It is expected that identification of such thermodynamic bottlenecks would be valuable to optimize the metabolism of *G. sulfurreducens *for practical application in microbial fuel cells (MFC). The rate of transfer of electrons to the electrode is directly linked to the metabolic rate. However, the rate of electron transfer is very slow in existing MFCs and it is possible that some of the limitations may be attributed to thermodynamic bottlenecks existing in the metabolic network of *G. sulfurreducens*. Thus, thermodynamics based modeling to determine potential bottlenecks will be valuable to prioritize candidates for metabolic engineering in order to increase flux through these target pathways. Hence, we anticipate that the modeling results will be ultimately beneficial to the improved characterization of the metabolism of *G. sulfurreducens *and optimization of power production in a MFC. Finally, in order to further constrain the metabolite activities and the Δ_r_G' based on thermodynamic feasibility constraints in the TMFA model, future efforts should focus on including experimentally measured large-scale metabolite concentrations coupled with kinetic constraints [15,16] to improve the predictive power of these genome-scale models.

## Competing interests

The authors declare that they have no competing interests.

## Authors' contributions

SG and RM conceived and designed the experiments. SG performed the experiments and generated the results. LY provided valuable insights and suggestions during execution of the experiments and helped in coding a part of the TMFA algorithm. SG and RM analyzed the data and wrote the manuscript. All authors read and approved the final manuscript.

## Supplementary Material

Additional file 1 **Supplemental table S1: List of reactions, metabolites in the metabolic network of *G. sulfurreducens *and their associated standard free energy change (kcal/mol)**. The list of standard free energy change associated with the metabolic reactions in *G. sulfurreducens *as determined by the group contribution method. Click here for file

Additional file 2 **Supplemental table S2: List of previously published microarray experiments utilized in this study**. A list of microarray experiments used in this study to investigate the change in the level of gene expression under varying environmental and genetic conditions for the model predicted reactions subject to regulatory control and thermodynamic bottlenecks. **Supplemental table S3**. Predicted regulatory sites and their corresponding ranges of gene expression in *E. coli*. A list of model predicted (gene-associated and a non gene associated) reactions that are subject to regulatory control in the metabolic network of *E. coli *and the expression ranges of the corresponding genes. **Supplemental table S4**. Bottleneck reactions and range of expression fold changes for the corresponding genes in *E. coli*. A list of model predicted (gene-associated and a non gene associated) bottleneck reactions in the metabolic network of *E. coli *and the expression ranges of the corresponding genes. **Supplemental table S5**. List of predicted strong candidate reactions subject to regulation, their associated genes and the minimum and maximum model predicted free energy changes (kcal/mol) in *G. sulfurreducens*. A list of model predicted (gene-associated and a non gene associated) reactions that are subject to regulatory control in the metabolic network of *G. sulfurreducens *and the lower and upper bounds of their Gibbs free energy change. **Supplemental table S6**. List of reactions at the threshold of being subject to regulatory control and their associated free energy change values (kcal/mol) in *G. sulfurreducens*. List of reactions in the *G. sulfurreducens *metabolic network whose upper bound on the Gibbs free energy change is in the vicinity of equilibrium and whose lower bound is far from equilibrium. **Supplemental table S7**. List of model predicted bottleneck reactions, their associated genes and the ranges in free energy under optimal growth using acetate as the electron donor and fumarate as the electron acceptor, in *G. sulfurreducens*. Analysis assuming no uncertainty in standard free energy change. Model predicted thermodynamically constrained (thermodynamic bottleneck) reactions that operate in the vicinity of equilibrium in the metabolic network of *G. sulfurreducens *and the associated lower and upper bounds of the Gibbs free energy change. Click here for file

Additional file 3 **Supplemental table S8: List of model predicted reactions in *G. sulfurreducens *and their corresponding gene expression ranges**. Gene expression ranges under various environmental and genetic perturbations filtered below a co-variance threshold for reactions subject to regulatory control and bottleneck reactions in *G. sulfurreducens*. Click here for file
