# Supplementary material for: Thermodynamic analysis of regulation in metabolic networks using constraint-based modeling
Source: BMC Res Notes. 2010 May 5;3:125. doi: 10.1186/1756-0500-3-125 (PMC2873351; doi:10.1186/1756-0500-3-125)
Supplement: Additional file 2 — Supplemental table S2: List of previously published microarray experiments utilized in this study. A list of microarray experiments used in this study to investigate the change in the level of gene expression under varying environmental and genetic conditions for the model predicted reactions subject to regulatory control and thermodynamic bottlenecks. Supplemental table S3. Predicted regulatory sites and their corresponding ranges of gene expression in E. coli. A list of model predicted (gene-associated and a non gene associated) reactions that are subject to regulatory control in the metabolic network of E. coli and the expression ranges of the corresponding genes. Supplemental table S4. Bottleneck reactions and range of expression fold changes for the corresponding genes in E. coli. A list of model predicted (gene-associated and a non gene associated) bottleneck reactions in the metabolic network of E. coli and the expression ranges of the corresponding genes. Supplemental table S5. List of predicted strong candidate reactions subject to regulation, their associated genes and the minimum and maximum model predicted free energy changes (kcal/mol) in G. sulfurreducens. A list of model predicted (gene-associated and a non gene associated) reactions that are subject to regulatory control in the metabolic network of G. sulfurreducens and the lower and upper bounds of their Gibbs free energy change. Supplemental table S6. List of reactions at the threshold of being subject to regulatory control and their associated free energy change values (kcal/mol) in G. sulfurreducens. List of reactions in the G. sulfurreducens metabolic network whose upper bound on the Gibbs free energy change is in the vicinity of equilibrium and whose lower bound is far from equilibrium. Supplemental table S7. List of model predicted bottleneck reactions, their associated genes and the ranges in free energy under optimal growth using acetate as the electron donor and fumarate as the electron acc [file 1756-0500-3-125-S2.DOC]

**Additional Files**

Additional File 1: Excel File 1: SITable1_Gs_Rxns_deltaG’s

Table 1: List of reactions, metabolites in the metabolic network of *G. sulfurreducens* and their associated standard free energy change (kcal/mol).

Additional File 2: This file.

Table 2: List of previously published microarray experiments for *G. sulfurreducens* utilized in this study.

| Microarray Experiment | Perturbation | Publication |
| --- | --- | --- |
| Fe Lim | Environmnetal | Mahadevan et al., (2008) |
| SfrAB | Genetic (Regulatory) |
| Fnr2^b^ | Genetic (Regulatory) |
| RpoE^b^ | Genetic (Regulatory) |
| Rel^GsU^ | Genetic (Regulatory) |
| Fum/Fe | Environmental |
| Fur | Genetic (Regulatory) |
| Oxygen | Environmental |
| OmcB | Genetic (Metabolic) |
| Biofilm | Environmental |
|  |  | Publication |
| RpoS | Genetic (Regulatory) | Krushkal et al., (2007)  Didonato et al., (2006),  Risso et al., (2008)  Holmes et al., (2006)  Juarez K et al., (2009)  Nevin et al., (2005)  Methe et al., (2005) |
|  |  |
| RelA,  Ac lim/Fum lim | Genetic(Regulatory), Environmental |
|  |  |
| Ele Fum, Ele Fe | Environmental |
|  |  |
| Fnr1,Fnr2 | Genetic (Regulatory) |
|  |  |
| Pi lim  Fe(III) reduction | Environmental  Environmental |

Table 3: Predicted regulatory sites and their corresponding ranges of gene expression in *E. coli*.

| Reactions subject to regulation in  *E. coli* | Associated Gene expression range  (fold change) |
| --- | --- |
| CS (gltA)  DHDPS^*^ (dapA)  CYSTL^*^ (metC)  ADCL^*^ (pabB)  ACLS^*^ (ilvB)  ME1x^*^ ( maeB)  PFK (pfkB)  G3PD (glpA)  ATPS (atpI) | -5.166 to 4.239  -2.28 to 2.18  -2.665 to 3.484  -3.482 to 1.563  -3.184 to 4.15  -3.02 to 2.817  -3.156 to 3.399  -3.35 to 5.67  -2.62 to 4.14 |

CS: citrate synthase, DHDPS: dihydrodipicolinate synthase, CYSTL:Cystathionine b-lyase, ADCL: 4-aminobenzoate synthase, ACLS: acetolactate synthase, ME1x:malic enzyme (NAD), PFK: Phosphofructokinase, G3PD: glycerol-3-phosphate dehydrogenase, ATPS: ATP synthase.

Table.4: Bottleneck reactions and range of expression fold changes for the corresponding genes in *E. coli*.

| Reactions operating close to equilibrium in  *E. coli* | Associated Gene expression range  (fold change) |
| --- | --- |
| MDH (scfA)  RPE (rpe)  PGI (pgi)  PGMT (pgm)  GAPD (gapA)  PGK (pgk) | -1.12 to 0.423  -1.346 to -0.427  -0.781 to -0.182  -1 to -0.716  -0.058 to 1.829  0.138 to 0.201 |

MDH: malate dehydrogenase, RPE: D-ribulose-5-phosphate 3-epimerase, PGI: phosphoglucose isomerase, PGMT: phosphoglucomutase, GAPD: glyceraldehyde 3-phoshpate dehydrogenase, PGK: phosphoglycerokinase.

*Table 5: List of predicted strong candidate reactions subject to regulation, their associated genes and the minimum and maximum model predicted free energy changes (kcal/mol) in G. sulfurreducens*.

| **Reaction** | **Metabolic Pathway classification** | **Associated gene** | **Minimum model predicted ∆_r_G’**  **(kcal/mol)** | **Maximum model predicted ∆_r_G’**  **(kcal/mol)** | **Model Predicted Nature** |
| --- | --- | --- | --- | --- | --- |
| ACHBS | Amino acid Metabolism | GSU1911,GSU1736,GSU1910 | -17.9 | -2.25 | Strong candidate |
| ACLS | Amino acid Metabolism | GSU1911,GSU1736,GSU1910 | -17.9 | -2.25 | Strong candidate |
| ADCL | Amino acid Metabolism | GSU0523 | -41.2 | -31.12 | Strong candidate |
| CS | Central Metabolism | GSU1106 | -10 | -9.2 | Strong candidate |
| DHDPS | Amino acid Metabolism | GSU0159 | -37.26 | -16 | Strong candidate |
| LDH_L | Central Metabolism | GSU1466 | -33.78 | -28.89 | Strong candidate |
| ME1X | Central Metabolism | GSU2308 | -4.63 | -4.45 | Strong candidate |
| PC | Central Metabolism | GSU2428 | -19.2 | -1e-06 | Strong  candidate |
| PPDK | Central Metabolism | GSU0580 | -28.03 | -2.53 | Strong candidate |
| SHCHCS2 | Vitamins and cofactor biosynthesis | Non-gene associated reaction | -50.21 | -29.87 | Strong candidate |

ACHBS: 2-aceto 2-hydroxy butanoate synthase, ACLS: acetolactate synthase, ADCL: 4-aminobenzoate synthase, CS: citrate synthase, DHDPS: dihydrodipicolinate synthase, LDH_L: lactate dehydrogenase, ME1X:malic enzyme (NAD), PC: pyruvate carboxylase, PPDK, phosphoenolpyruvate dikinase, SHCHCS2: 2-succinyl-6-hydroxy-2,4-cyclohexadiene 1-carboxylate synthase.

Table 6: List of reactions at the threshold of being subject to regulatory control and their associated free energy change values (kcal/mol) in *G. sulfurreducens.*

| Reaction | Metabolic Pathway classification | Associated gene | Minimum model predicted ∆_r_G’  (kcal/mol) | Maximum model predicted ∆_r_G’  (kcal/mol) | Model Predicted Nature |
| --- | --- | --- | --- | --- | --- |
| CYSTL | Amino acid metabolism | GSU0944, GSU0945 | -10 | 0.72 | Threshold of regulation |
| PPS | Central Metabolism | GSU0803 | -22.21 | 3.013 | Threshold of regulation |
| DXPS | Vitamins and cofactor biosynthesis | GSU1764, GSU0686 | -15.7 | 0.047 | Threshold of regulation |

CYSTL:Cystathionine b-lyase, PPS:, phosphoenolpyruvate synthase DXPS: 1-deoxy-D-xylulose 5-phosphate synthase.

Table 7: List of model predicted bottleneck reactions, their associated genes and the ranges in free energy under optimal growth using acetate as the electron donor and fumarate as the electron acceptor, in *G. sulfurreducens.* (Analysis assuming no uncertainty in standard free energy change.)

| **Reaction** | **Metabolic Pathway classification** | **Associated gene** | **Minimum model predicted ∆_r_G’**  **(kcal/mol)** | **Maximum model predicted ∆_r_G’**  **(kcal/mol)** | **Model Predicted Nature** |
| --- | --- | --- | --- | --- | --- |
| FUM | Central Metabolism | GSU0994 | -1e-06 | -1e-06 | Bottleneck |
| ADSL1 | Nucleotide Metabolism | GSU1632 | -0.179 | -1e-06 | Bottleneck |
| ADSL2 | Nucleotide Metabolism | GSU1632 | -0.179 | -1e-06 | Bottleneck |
| MDH | Central Metabolism | GSU1466 | -4.70 | -1e-06 | Bottleneck |
| ME2 | Central Metabolism | GSU1700 | -4.5 | 5.67 | Bottleneck |
| ASPTA1 | Nucleotide Metabolism | GSU1061,GSU1242 | -4.68 | 5.8 | Bottleneck |
| ALAD_L | Amino acid Metabolism | GSU2292 | -4.33 | 5.7 | Bottleneck |

FUM: fumarase, ADSL1: adenylsuccinate lyase, ADSL2: adenylosuccinate lyase, MDH: malate dehydrogenase, ME2: malic enzyme (NADP), ASPTA1: aspartate transaminase, ALAD_L: L-alanine dehydrogenase.

Additional File 3:

Supplementary Excel File 2: SITable8_Gs_Exp_Ranges

Table 8: List of model predicted reactions in *G. sulfurreducens* and their corresponding gene expression ranges
